# Preliminary Evidence for a Window of Increased Vulnerability to Sustain a Concussion in Females: A Brief Report

**DOI:** 10.3389/fneur.2019.00691

**Published:** 2019-07-09

**Authors:** Michael F. La Fountaine, Vicci Hill-Lombardi, Asante N. Hohn, Caroline L. Leahy, Anthony J. Testa

**Affiliations:** ^1^Department of Physical Therapy, School of Health and Medical Sciences, Seton Hall University, Nutley, NJ, United States; ^2^The Institute for Advanced Study of Rehabilitation and Sports Science, Seton Hall University, Nutley, NJ, United States; ^3^Department of Medical Sciences, Hackensack Meridian School of Medicine at Seton Hall University, Nutley, NJ, United States; ^4^Department of Neurology, Hackensack Meridian School of Medicine at Seton Hall University, Nutley, NJ, United States; ^5^Department of Athletic Training, School of Health and Medical Sciences, Seton Hall University, Nutley, NJ, United States; ^6^Center for Sports Medicine, Seton Hall University, South Orange, NJ, United States

**Keywords:** menstrual cycle, luteal phase, concussion, brain injury-traumatic, womens health

## Abstract

A difference exists between sexes for the incidence of concussion injuries and severity of post-injury outcomes with females having a higher incidence rate (in comparable sports) and experience more robust symptoms than males. The basis for this disparity has remained largely unresolved. Recent findings point to a potential biological mechanism that may be related to the menstrual cycle as an arbiter of post-injury outcomes. What has not been addressed, is whether the phase of menstrual cycle (inferred fluctuations of ovarian hormones) contributes to an increased vulnerability to sustain a concussion injury. This prospective, observational study sought to determine if concussions occurred at different frequencies throughout the phase of the menstrual cycle. Female athletes who sustained a concussion injury were queried three times over the 7-day study (e.g., within 48 h of injury, and 4 and 7 days after injury) to recall the number of days that have elapsed since the beginning of their most recent menstruation. Twenty female athletes enrolled after sustaining a concussion; 18 were eumenorrheic and 2 amenorrheic. Among eumenorrheic participants at the time of injury, 2 were in the follicular phase, 4 were in the early luteal phase and 9 were in the late luteal phase. Two athletes were injured on the first and 1 was injured on the second day of menstruation. The greatest number of concussions were sustained during the late luteal phase and during the first 2 days of menstruation. This 9-day window accounted for 2/3rd of the sustained concussions in our study.

## Introduction

A longstanding discussion in the field of concussion injury and management centers on whether a difference exists between sexes for the incidence of injury and severity of post-injury outcomes (1–6). In short, does the athlete's sex contribute to the risk for sustaining a concussion when participating in similar sports, or exaggerate symptoms and recovery following an injury? From a pure incidence perspective, epidemiological studies report that females (e.g., high school and college-aged athletes) have a higher rate of concussion injuries than males in comparable sports (5, 7). In high school aged girls, the overall concussion rate per 10,000 athlete exposures, defined as one athlete participating in one practice or competition, was 2.64 (95% confidence interval: 2.37, 2.90) compared to 1.69 (1.51, 1.88) in boys competing in similar sports (7). In college-aged athletes, the respective concussion rate per 1,000 athlete exposures was consistently higher in females than males in basketball [e.g., 0.53 (0.43, 0.64) vs. 0.38 (0.29, 0.46)], lacrosse [e.g., 0.45 (0.32, 0.57) vs. 0.30 (0.22, 0.39)], and soccer [e.g., 0.54 (0.43, 0.64) vs. 0.26 (0.17, 0.35)], respectively, (5).

The presentation and range of symptomatic experiences after a concussion are well-documented and include potential impairment to affect (8–11), cognition (12–15), sleep (16–18), vesitbular-occulomotor (19–21), and cardiovascular autonomic function (22, 23). The challenge for clinicians has been a limited ability to predict the type, number, and severity of symptoms that may emerge, and equally important, how long they persist after a concussion. A snapshot of evidence suggests that females are more likely than males to present with reduced performance on visual memory tasks (3), experience more discomfort after injury, and are more likely to seek treatment for post-concussion headaches (24), while other studies have shown no differences in symptom burden between males and females (3, 25–27). But, the question remains as to why an applied mechanical force to the head or body is more likely to cause a concussion or lead to differing post-injury symptoms in females vs. males?

A few recent reports (28, 29) demonstrated that a biological basis for these observations may arise from the menstrual cycle as a potential arbiter for the post-injury symptomatic experience from concussion. In one study (29), women who sustained a mild traumatic brain injury (MTBI) during the luteal phase of their menstrual cycle had a significantly lower quality of life and indicators of health 1 month after discharge than those females who were in the follicular phase of the cycle or were taking hormone contraceptives. In a separate study (28), authors found that males tended to recover from injury more quickly than females and that the initial symptom severity after concussion in male athletes was correlated to the length of recovery. In females, however, the symptom burden after concussion was more closely associated to the use of hormone contraceptives. In other words, females, who were not taking hormone contraceptives at the time of injury experienced more severe symptoms compared to those taking hormone contraceptives. Thus, a differential in effective hormone concentrations at the time of injury partially contributed to the post-injury symptomatic experience in females who were taking hormone contraceptives vs. those who were not. But, what has not been addressed, is whether the inferred changes in circulating hormone concentrations throughout the menstrual cycle, and its respective phases, contribute to a difference in the frequency of concussion injuries that occur in female athletes. The purpose of this study was, therefore, to document the frequency of concussion injuries across the respective phases of the menstrual cycle (e.g., menstruation, follicular phase, early luteal phase, and late luteal phase) in college-age female athletes. Based on the available evidence, we hypothesized that the greatest number of concussions would be sustained during the late luteal phase when hormone concentrations that are speculated to impart neuroprotective effects are anticipated to be on the decline to their monthly nadir.

## Materials and Methods

**Figure 1:**
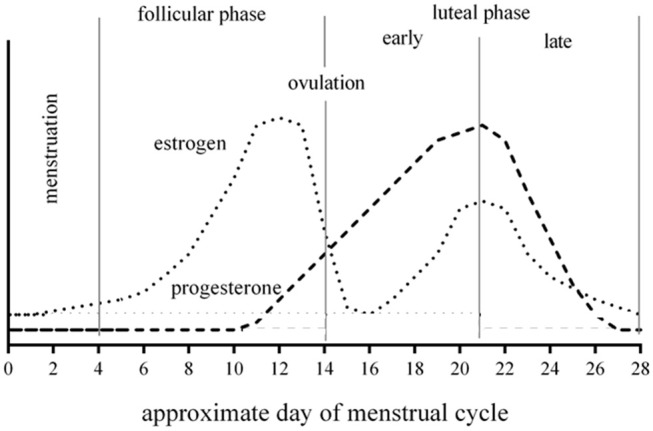
Example 28-day menstrual cycle with changes of ovarian hormones during respective phases.

A prospective, cross-sectional institutional review board (IRB) approved study, which is part of a larger on-going initiative with separate outcome measures, was performed in female collegiate athletes aged 18–22 years old. A player suspected of sustaining a head injury was evaluated using clinically accepted practices of concussion assessment for both immediate on-field and subsequent office-based follow-up evaluations. Prospective participants were alerted to the existence of the research study through IRB approved means including recruitment flyers and word-of-mouth referrals. Upon initial contact between the researchers and the prospective participant, the study aims and expectations of participating were described. If, at that time, there were any questions, they were addressed. If not, a study visit was scheduled and written informed consent was obtained from all participants prior to initiating the study procedures. To be eligible for study participation, the concussed athlete must have sustained a concussion within the previous 48 h, demonstrate capacity to provide written informed consent, not be taking medications or substances with direct or indirect actions on the cardiovascular or central nervous systems, and be free of acute illness or trauma that would otherwise preclude their participation. During the intake examination at each of 3 study visits occurring within the first post-injury week after sustaining a concussion (e.g., within 48 h of injury/symptom presentation, 4 and 7 days later), participants were asked to indicate the number of days since the beginning of their most recent menstruation. All participants provided the start date of the most recent menstruation after reviewing a calendar, by immediate recall, or in some cases, an athlete referred to an application on her personal smart phone specific to tracking menstrual function/ patterns. Each athlete provided the identical dates for the start of their most recent menstrual cycle. The participants were also asked to provide their age of menarche, use/non-use of hormone contraceptives and characterize their recent menstrual cycle history (e.g., prior 3 months) as being regular (eumenorrhea: ~1 cycle per month), or irregular (amenorrhea: absence of menstruation; oligomenorrhea: >35 to <90 days between successive cycles). For the purposes of this report, a normal menstrual cycle was defined as 28 days and the phases were standardized: menstruation (0–4 days); follicular phase (5–14 days); early luteal phase (15–21 days); and late luteal phase (22–28 days) (Figure 1). As part of the research intake, participants were asked to recall their past concussion history and were evaluated at each visit by the same rater with the Sport Concussion Assessment Tool (SCAT) version 3 (SCAT 3) (30) to characterize their post-injury affect and symptom presentation. Due to the descriptive nature of this report and small sample sizes, statistical analyses were not performed.

## Results

As of this report, 20 female athletes enrolled in our study after sustaining a concussion; 18 were eumenorrheic and 2 were amenorrheic. Of the eumenorrheic females, 12 participants reported that they did not take hormone contraceptives, while 6 reported that they took oral hormone contraceptives (Table 1). All eumenorrheic participants reported having a routine, predictable monthly cycle in the months preceding their enrollment in the study. Two athletes taking hormone contraceptives were found to be amenorrheic; upon further discussion it was revealed that this was a voluntary decision to continuously suppress ovulation. Table 1 provides information about the participants including the phase of menstrual cycle, use/non-use of hormone contraceptives, number of past diagnosed concussions and the SCAT 3 symptom score for each visit. Specific demographic detail and sports played are not included to maintain the confidentiality of the athletes in this small cohort study.

**Table 1:** Characteristics of study participants.

**Subject #**	**Phase of menstrual cycle upon injury**	**Use of hormone contraceptives**	**Age of menarche**	**# of past diagnosed concussions**	**SCAT 3 symptom score visit 1**	**SCAT 3 symptom score visit 2**	**SCAT 3 symptom score visit 3**
1	ELP	No	12	1	48	51	34
2	FP	Yes	13	1	5	0	0
3	MP	Yes	13	2	30	26	13
4	LLP	No	10	3	19	17	16
5	LLP	No	11	1	51	12	0
6	LLP	No	14	0	55	29	10
7	MP	No	12	0	20	0	0
8	FP	No	12	3	18	0	0
9	LLP	No	13	2	13	3	7
10	LLP	No	13	0	36	10	8
11	Amenorrhea	Yes	13	1	41	18	3
12	Amenorrhea	Yes	11	0	29	32	11
13	ELP	No	12	1	23	12	6
14	LLP	Yes	15	0	32	16	23
15	ELP	Yes	13	5	6	4	0
16	MP	No	14	2	36	22	5
17	LLP	No	12	2	65	41	24
18	LLP	No	14	0	8	0	0
19	LLP	Yes	12	3	33	25	26
20	ELP	Yes	12	0	24	12	8

*ELP, early luteal phases; FP, follicular phase; LLP, late luteal phase; MP, menstrual phase; SCAT, sport concussion assessment tool*.

Upon review of the data, it was revealed that 3 participants sustained their injury on the first or second day of menstruation (1 taking hormone contraceptives), 2 participants were in the follicular phase (1 taking hormone contraceptives), 4 participants were in the early luteal phase (2 taking hormone contraceptives), and 9 participants were in the late luteal phase (2 taking hormone contraceptives) at the time of injury. In total, 9 of 18 (50%) eumenorrheic participants sustained their concussion during a 7-day window characterized by the late luteal phase and no other phase exceeded a 22% injury rate. We hypothesized that the occurrence of concussion injuries would be the greatest when hormone concentrations were expected to be on the decline or were at their monthly nadir. In this context and in consideration for the timing of the concussion injuries early in the menstrual phase, it may be argued that when including these 3 injuries to the 9 from the late luteal phase, 66.7% (12/18 eumoenrrheic athletes) of the concussions occurred during a narrow 9-day window. There was a wide range of early symptom severity, but no emerging trends as to whether the symptom burden was attributable to the phase of menstrual in this small cohort. Similarly, all but 6 participants had a past history of at least 1 diagnosed concussion injury. Because the composition of injury occurrence varied during the menstrual phase in our cohort of concussed female athletes, the use of statistics to evaluate the potential role for past concussion history on our outcomes would be inappropriate. However, as presently constituted, the average number of past concussions for each cohort are provided: late luteal phase = 1.2; menstrual phase = 1.3; early luteal phase = 1.8; and, follicular phase = 2.0.

## Discussion

This preliminary observation demonstrates that 50% of concussions experienced in our cohort of college-aged female athletes occurred during a 7-day window characterized by the late luteal phase. If including cases where females sustained their concussion on the first or second day of menstruation, 66.7% of concussions happened during a 9-day window. The significance of this window of time, is that the hormones estrogen and progesterone are declining to, or are at their monthly nadir. For those taking oral hormone contraceptives, this window of time corresponds to when the placebo pill is often taken (or skipped), and as such, there is no exogenous support of circulating hormone concentrations.

The role of menstrual cycle phase at the time of MTBI was explored by Wunderle and colleagues who followed 144 females (16–60 years old) for 1 month after emergency department discharge (29). The investigators found that women who sustained a MTBI during the luteal phase of injury (*n* = 37) had a significantly lower quality of life and indicators of health 1 month after discharge than those females who were in the follicular phase of the cycle (*n* = 72) or were taking hormone contraceptives (*n* = 35). To describe their 1-month post-injury observation of more adverse outcomes in women who sustained a MTBI during the luteal phase despite a presumed increase in circulating progesterone concentrations at the time of injury, Wunderle et al. proposed the “withdrawal hypothesis” (29). Specifically, the hypothesis stated that a “…TBI occurring in the setting of high progesterone results in a sudden decrease of progesterone.” The basis for the decline in progesterone among their luteal phase injury participants was not specified, nor was a blood concentration obtained to verify it. However, secondary hypopituitarism has been shown to emerge following MTBI across a wide range of clinical presentations (31–37). In their study cohort, nearly twice as many females who met the inclusion criterion sustained the MTBI during the follicular phase than in the luteal phase, and women taking hormone contraceptives were separated into their own group without consideration for the phase of the cycle that they were in (e.g., where they were in the pill pack), if they had a monthly menstruation or were suppressing menstruation altogether. This latter observation challenges our hypothesis for the presence of a narrow window of increased vulnerability to sustain a concussion during the menstrual phase. However, it should be noted that the etiology for the MTBI in their participants was not disclosed (e.g., motor vehicle accident, fall, occupational, sports, violence, etc.). And, unlike organized sporting activities where any suspected head trauma is promptly evaluated by a qualified healthcare professional within the university setting, the experience of an injury occurring under free-living conditions in the community may result in individuals not seeking medical attention, or going to a provider/facility other than where the research study was performed. As a result, there are challenges in comparing the inclusive findings to that from the Wunderle study.

In a report by Gallagher et al. entitled, “The Effects of Sex Differences and Hormonal Contraception on Outcomes after Collegiate Sports-Related Concussion,” the authors demonstrated that males tended to recover from injury more quickly than females and that the initial symptom severity after concussion in male athletes was correlated to the length of recovery (28). In females, however, the symptom burden after concussion was more closely associated to the use of hormone contraceptives such that females who were not taking hormone contraceptives at the time of injury experienced more severe symptoms compared to those taking hormone contraceptives. The post-injury consequences of concussion injuries on menstrual cycle dysregulation were recently highlighted in an article by Snook et al. (38) entitled “Association of Concussion with abnormal menstrual patterns in adolescent and young women.” The authors demonstrated that ~1 in 4 females (12–21 years old) with concussion experienced at least 2 abnormal menstrual patterns within the first 120 days after injury, compared to only 5% of participants with non-head sport-related orthopedic injury; this disparity contributed to a significantly elevated odds ratio of 5.85 (95% confidence interval: 1.61–21.22) for the occurrence of menstrual dysfunction after concussion injury. The collection of evidence highlights an interesting inter-related paradigm: hormones that regulate the menstrual cycle and their circulating concentrations at the time of head injury appear to influence the post-injury symptomatic experience; a head injury may influence the post-injury secretory patterns of hormones that regulate the menstrual cycle leading to an increased risk of dysfunction in subsequent months; and, with our findings, concussion injuries appear to occur more frequently during the phase of the menstrual cycle commonly associated with declining or low estrogen and progesterone concentrations.

In addition to having a very specific and potent effect on the uterus, ovarian hormones (e.g., estrogen and progesterone) are demonstrated to exert neuroprotective effects (39–42). Estrogen and progesterone-mediated neuroprotection is thought to be related to their effects on hormone receptors, direct antioxidant effects, effects on astrocytes and microglia, modulation of the inflammatory response to injury, and effects on mediating glutamate excitotoxicity, among others (40–42). Neuroprotection by testosterone has also been explored, and appears to be related to its action on androgen receptors (43–45). However, neuroprotection by testosterone has been suggested to be difficult to directly study because the hormone can aromatize to estrogen through the action of enzymes (46). Because testosterone concentrations in females are generally about 10% or less than that of males, the focus of hormonally-mediated neuroprotection will focus on estrogens and progesterone. The luteal phase of the ovarian cycle is characterized by two distinct patterns of change in estrogen and progesterone concentrations; in the early phase after ovulation, these hormones ascend to their peak concentration (during the phase), and then steadily decline through the late phase until the initiation of menstruation where they stay low for the first few days (estrogen) or ~10 days (progesterone).

If we appreciate the time-course of progesterone secretion as part of the normal menstrual cycle and our 66.7% incidence rate of concussion during the late luteal phase and early menstruation, it stands within reason to expect that progesterone concentrations would continue to fall or remain low for an ~2–3-week window after injury. Under these circumstances, the ability to mobilize and exploit neuroprotection (40–42) or repair via increasing estrogen or progesterone concentrations may be constrained because of the intrinsic secretory patterns that regulate the menstrual cycle or the potential for hypothalamic-pituitary-gonadal axis impairment following concussion. For those taking oral hormone contraceptives, the initiation of the next cycle of pills would exogenously support hormone concentrations, and perhaps facilitate changes to ameliorate symptoms. As such, these circumstances and the potential role(s) of ovarian hormones in recovery from injury may have contributed to the protracted symptoms and reductions to quality of life and health described by Wunderle et al. (29). This position may also assist in explaining the past observation of females who were not taking hormone contraceptives at the time of injury experiencing more severe symptoms compared to those taking hormone contraceptives (28).

Caution should be exercised against presuming that increasing concentrations of estrogen and/or progesterone through exogenous supplementation would facilitate healing, or recovery or management of post-injury symptoms, as there is no evidence to suggest that such an intervention would be warranted, unless, of course, the presence of a clinically-significant hormone deficiency has been identified after screening by an endocrinologist. In addition, the synthesis of this collective evidence does not mean that higher ovarian hormones are prophylactic against sustaining a concussion or attenuating the post-injury symptomology because we know that injuries can occur at any time. At this time, one can only conclude that that lower hormone concentrations that may be associated with the late luteal phase or early menstruation appears to create a window of vulnerability where females sustain concussion at a greater rate than other intervals during the menstrual cycle.

There are several limitations from this preliminary analysis of an on-going study. First, we are reporting outcomes from a small cohort of female athletes who participated in varsity athletics and sustained an injury at a single site. Second, we are working under the assumption that each eumenorrheic female had a consistent 28-day cycle. Third, we did not obtain blood samples to support the narrative of the potential role for low hormone concentrations. Finally, we relied on the participant's self-reported accounting for the number of days since their last cycle; to minimize bias, we asked each participant to recall the date at each visit without prompting for the information previously provided. There was only 1 participant who sustained a concussion on a transitional day (e.g., last day of one cycle) for the phase of the cycle; in this case, she sustained the injury 14 days since the first day of her most recent menstruation and is described as being in the follicular phase in our dataset.

In conclusion, there appears to be a 9-day window of increased vulnerability to sustain a concussion during the late luteal phase of the menstrual cycle and the first few days of menstruation. This risk may be associated with declining or low concentrations of estrogen and progesterone that could be speculated to reduce neuroprotection, and perhaps, blunt the recovery time from symptoms. Because of the inferred reduction to neuroprotection as a result of the declining hormone concentrations during these phases of the menstrual cycle, we speculate that females participating in sporting activities during this epoch of time may be more vulnerable to sustain a concussion injury when exposed to a diverse magnitude of applied mechanical forces to the head and/or body compared to other phases where circulating hormone concentrations may be higher and impart greater neuroprotection. Whether or not these hormonal changes contribute to the differences in concussion between sexes previously described for the incidence and severity of outcomes remains to be seen. In order for the field to develop a substantive base of knowledge on this topic, a more comprehensive study is needed and should include comparisons of the frequency of injuries across the phases of menstrual cycle to other forms of traumatic sports injury (e.g., soft-tissue tear and/or rupture, fractures, etc.) and what role, if any, past concussion history, or use/type of hormone contraceptive (e.g., oral, implant, injection, etc.) has on the reported outcomes. To facilitate this narrative, stakeholders responsible for clinical surveillance or those engaging in prospective research should consider incorporating the following measurements to their prospective practices and observations:

1. Screen for health/behavior indicators that may negatively impact eumenorrhea,
2. Ask females to disclose their use/non-use of hormone contraceptives during the pre-participation physical examination and alert clinical staff to changes in their medication(s),
3. Ask females who sustain a concussion to disclose the first day of their last menstruation, and,
4. Follow-up with and document menstrual cycle patterns (e.g., time of arrival, length of cycle, changes to flow/discharge of fluids, associated symptoms) for several cycles after a concussion injury.

## Data Availability

The data supporting the findings presented in this study are available within the article. Because the primary study is on-going, data will not be shared at this time.

## Ethics Statement

This study was carried out in accordance with the recommendations of name of guidelines, name of committee with written informed consent from all subjects. All subjects gave written informed consent in accordance with the Declaration of Helsinki. The protocol was approved by the name of committee.

## Author Contributions

ML: conceptualization, funding acquisition, formal analysis, and writing original draft. ML, VH-L, AH, CL, and AT: investigation and writing, review and editing, formal approval of final draft. ML and AT: methodology. ML, VH-L, AH, and CL: project administration. ML, VH-L, and AT: supervision.

### Conflict of Interest Statement

The authors declare that the research was conducted in the absence of any commercial or financial relationships that could be construed as a potential conflict of interest.
